# Compliance with standard safety precautions and associated factors among health care workers in Hawassa University comprehensive, specialized hospital, Southern Ethiopia

**DOI:** 10.1371/journal.pone.0239744

**Published:** 2020-10-15

**Authors:** Tsegaye Bekele, Tesfaye Ashenaf, Adane Ermias, Abinet Arega Sadore

**Affiliations:** 1 Sidama Zone Health Department, Hawassa, Ethiopia; 2 Department of Environmental Health, Hawassa University, Hawassa, Ethiopia; 3 Wachemo University School of Public Health, Hossana, Ethiopia; Centre de Recherche en Cancerologie de Lyon, FRANCE

## Abstract

**Background:**

Globally, health care-associated infections had become serious public health importance. Compliance with standard safety precaution is effective and inexpensive measure to improve quality of healthcare in reducing occurrence of healthcare associated infections. In developing countries, like Ethiopia adherence to recommended standard safety precaution is scanty.

**Objective:**

To assess level of compliance with standard safety precaution and associated factors among healthcare workers in Hawassa comprehensive specialized hospital Southern Ethiopia.

**Methods:**

An institutional based cross-sectional study was conducted at Hawassa comprehensive specialized hospital. Data were collected by using self-administered questionnaire. Study participants were allocated proportionally based on their profession by using stratified random sampling method. Data were entered and analyzed by using SPSS version 20.0. Bi-variable analysis and multi variable logistic regression model were used to check which variables were associated with dependent variable. P-values ≤ 0.05 were considered statistically significant. In this study the overall compliance with standard safety precaution among healthcare workers were only 56.5%. Being female healthcare worker AOR: 2.76(1.34, 5.54), married healthcare workers AOR: 4.2(2, 9.03), accessibility of safety box AOR: 3.4(1.6, 7.17), HCWs had perceived IP training AOR: 3.99(1.46, 10.9), availability of tape water AOR: 2.68(1.15, 6.2) and healthcare workers had internal infection prevention and control supportive supervision AOR: 5.8(2.54, 13.48) associated with compliance with standard safety precaution.

**Conclusion:**

According to findings of the current study, overall level of compliance with standard SP among HCWs considered to be very low. Factors such as healthcare workers being female, accessibility of safety box, availability of running tape water, training and supportive supervision were independent predictors of compliance with standard safety precaution. Thus ensuring availability and accessibility of safety precaution materials and regular observing and supervising healthcare workers’ practices are highly recommended.

## 1. Introduction

Health care-associated infections (HCAIs) are those infections that patients acquire while receiving health care. Globally, healthcare associated infections (HCAIs) become serious public health importance [1, 2]. HCAIs affect hundreds of millions of patients and about three million health care professionals around the world every year irrespective of their economic level of countries [3]. Accordingly as the European Center for Disease Prevention and Control (CDC) reports showed that prevalence of HCAIs ranges between 4.6%-9.3% in European countries and also the center reported that five million patients develop infections as result of healthcare in Europe; contributing for 135,000 deaths per year. In addition to this, United States of America (USA) estimated incidence rate of 4.5% in 2002, which accounts 1.7 million patients affected; from which 99,000 deaths per year as result of HCAIs [1].

In order to improve the wellbeing of patients, visitors, attendants, supportive staff, health care workers (HCWs) and general community in healthcare facilities; WHO developed different programs such as “Clean Care is Safer Care” as primary focus to promote hand hygiene practices globally at all level of healthcare as initial step in ensuring high level of infection prevention practices and control [3, 4]. Federal ministry of health (FMOH) has developed a number of guidelines in hospitals for infection prevention practices and control as effective, efficient and quality measures. Additionally, they obligate hospital administration to have strong infection prevention committee and environmental health officers since 2004 [5–7]. Hence in order to improve compliance with standard precautions (SPs) among HCWs; In Ethiopia different strategic intervention have been performing till now [5, 6] However, in most of the hospitals of Ethiopia there are guidelines, policies and laws of infection prevention practices and control, such as hand hygiene, personal protective equipments (PPEs), disinfection and sterilization, injection safety and proper waste management. Those all constituents of standard SPs are challenged by accessibility and availability of infrastructures, under staffing, shortage of basic PPEs, workload, inadequate structural organization and lack of awareness and on infection prevention practices and control guidelines [15].

Pertaining to Hawassa comprehensive specialized hospital there is problem of HCAIs even though the evidences are not well established and reported about HCAIs and as well as level of compliance with standard precautions.

Compliance with standard SPs is fundamental to quality of care, and essential to protect HCWs, patients, and communities [7]. Particularly in Ethiopia, where poor-resource; the prevalence of serious transmitting infectious diseases such as Hepatitis B, C and HIV is very high. And also preventive interventions for all these diseases are minimal [8]. Many of the pathogens that cause HCAIs can present and live on health care equipment, unwashed soiled hands and on healthcare setting environment in which patients and HCWs touch. These pathogens are easily transmitted from patient to patient or HCWs and vice versa that leads to serious morbidity, increase hospitalization days for patients, increased antimicrobial resistance, long term disability, high economic costs for patients and healthcare systems and tragic loss of life. Mainly as a result of healthcare professionals and facilities not follow prescribed IPPC [9, 10].

In 2010 Ethiopia hospitals reform implementation guidelines (EHRIG) and 2014 clean and safe health (CASH) initiatives had been launched in line with Ethiopian hospitals alliance for quality (EHAQ) which has the same aim on SPs. So that the main aim were to make hospitals clean, comfortable and safe environment for patient, attendants, visitors, staff and number of general public and to increase patient confidence and organizational commitment to assure patient safety and good health outcomes [2, 6]. Hawassa comprehensive specialized Hospital is working by integrating IPC programs with other relevant programs as a key intervention to ensure implementation of IPC policies and strategies in the hospital and to assure patient safety and good health outcomes.

However, Ethiopian hospitals have been implementing all the guidelines and policies in hospitals, still preventing and controlling risk of HCAIs is questionable for HCWs [11]. On behalf of this, there are multiple factors that standard SPs are always not used by HCWs as the different literatures had revealed, such as; unavailability and inaccessibility of PPEs; inadequate knowledge about standard SPs, less attitude towards standard SPs, low risk taking personality, less perception about efficacy of prevention, perception of risk and; less management support for safe work practice; safety performance feedback, work place safety, assigned place of work, work category and marital status of HCWs [6, 12, 13].

Generally, lack of compliance with SPs among HCWs has surplus consequences in our country in general and in Hawassa specifically leading to serious morbidity and mortality on the HCWs, patients and the community even if it is not well established and reported. And also there are insufficient studies are found regarding to the compliance with SPs and associated factors among HCWs in the country as well as Hawassa city. So that, the main aim of this study is assessing level of compliance with SPs and associated factors among HCWs in the Hawassa University Comprehensive Specialized Hospital, Southern Ethiopia.

## 2. Objectives

### 2.1. General objectives

To determine level of compliance with standard precautions and associated factors among healthcare workers in Hawassa comprehensive specialized hospital Southern Ethiopia Hawassa, 2019.

### 2.2. Specific objectives

To determine level of compliance with standard precautions in Hawas Comprehensive specialized Hospital, Southern Ethiopia, 2019.

To identify factors that influence compliance with standard precautions among health workers in Hawassa Comprehensive Specialized hospital, Southern Ethiopia, 2019.

## 3. Methodology

### 3.1. Study area and period

The study was carried out in Hawassa University comprehensive specialized hospital in Hawassa city, southern Ethiopia. It is capital city of Sidama Zone and Southern Nations, Nationalities People Regional states. Hawassa University Comprehensive Specialized, treating and teaching hospital is found in Hawassa city, which has more than 480 beds which provide service for about 18 million the SNNPR and neighboring Oromia region people in the area. The daily outpatient flow is more than 300 per day. This study was conducted from January 15 up to June 15, 2019.

### 3.2. Study design

An institutional based cross-sectional study was conducted in Hawassa Comprehensive specialized Hospital.

### 3.3. Source population

All health care workers.

### 3.4. Study population

All randomly selected study participants from all healthcare workers.

### 3.5. Inclusion and exclusion criteria

#### 3.5.1. Inclusion criteria

All health care workers who were involved in clinical services during study period and had direct contact with patient care including, residency medical training and intern medical students were included in the study.

#### 3.5.2. Exclusion criteria

The workers who were on annual and maternity leave during data collection, those who couldn’t respond to the questions due to illness and those working in administration offices were excluded from the study.

### 3.6. Sample size

The sample size was determined by using single population proportion formula by taking value of 50% (0.5) study conducted in Mekele special Zone by considering confidence interval (CI) 95%, margin of error 5% and using 10% for non-response rate (23). n was calculated by using Cochran formula, d, and adding 10% non-respondents (34).

$$\begin{array}{c} \frac{{\left(1.96\right)}^{2}*0.5*(1–0.5)}{{\left(0.05\right)}^{2}}\\ =384+(384*10\%)(\text{Non-response}\;\text{rate})\\ =422, \end{array}$$

Where, n = sample size; P = proportion Zα/2 = at 95% CI which is equal to 1.96

d = margin of error or desired precision which is equal to 5% = 0.05 After that 10% non-response rate; total sample sizes was 422.

### 3.7. Sampling procedures

Stratified random sampling method was used to select study participants. Health care workers were categorized according to their profession and then separate sample was taken based on an equal and independent chance for all HCWs appearing in the sample by using simple random sampling method. Stratified random sampling method was used to select 31.1% of HCWs from each category. The respondents were selected using computer generated random number.

### 3.8. Data collection procedure

The questionnaire was initially developed in English by reviewing available literatures and guidelines. Three trained Bsc Nurses collected data and two supervisors were employed to follow up the data collection process. Pretested structured self-administered questionnaire adopted from different literatures and Ethiopia IPC guidelines were used [6, 7]. Totally, the data collection tool included 23 compliance with standard SPs which were measured by five points likert scale questionnaires (1 = never, 2 = seldom, 3 = sometimes, 4 = often, 5 = always); 6 socio-demographic questions; 23 yes/no questions.

### 3.9. Data quality control

In order to assure data quality detail description in each question of questionnaire training was provided for the data collectors and supervisors. Questionnaires prepared in English language translated in to Amharic. The Amharic version was translated back into English by a researcher conversant in both languages. The two versions were examined to identify any inconsistency in the wording. From the total sample size 10% samples were used for pretesting tools and checked for validity and reliability as well as for ambiguity of questions among data collectors and supervisors. Chronbach’s alpha coefficient was used to estimate the reliability of questionnaire. Accordingly, pretesting was performed in Hawassa Adare General Hospital. Daily close supervision and data collectors for completeness and accuracy of collected data were checked.

**Dependent variables:** Compliance with standard safety precaution.

**Independent variables:** Socio-demographic factors**,** Personal factors and environmental factors.

#### 3.9.1. Operational definition

Compliance with standard safety precaution is complete following and practice of prescribed IPPC in work place all the time and for all patients; such as hand hygiene, always utilization of PPEs whenever necessary according to standard, sharp materials safety practices, sterilization and disinfection of instruments, healthcare waste management, availing policies and guidelines in working classes and training for HCWs.

#### 3.9.2. Data processing and analysis

Data collected from the respondents entered, cleaned and analyzed by using SPSS version 20.0 software package for further statistical analysis. Determinants of compliance with standard SP were explained by descriptive statistics, such as: frequency distribution, measures of central tendency, measures of variation and percentages displayed by using figure and tables to describe study subjects. Bi-variable analysis was used primarily to check which variables were associated with dependent variable individually. To limit the number of variable and unstable estimates in the subsequent models, only P-value <0.25 in the bi-variable further entered in to multi-variable logistic regression model. Bi-variable analysis was conducted to examine the various associations using chi-square test. The results presented by using crude and adjusted Odds ratios (ORs) and Confidence Interval (CI) 95%. P-values ≤ 0.05 were considered statistically significant and presented by adjusted odds (AOR) ratio with 95% confidence interval.

### 3.10. Ethical consideration

The research topic and methodology were approved by the ethical review committee of Hawassa University College of medicine and health science. Permission to conduct the study obtained from the Hawassa comprehensive specialized Hospital. The research presents no more than minimal risk of harm to subjects. Thus, oral consent was obtained from all the respondents after explaining of the purpose of the study, risk/discomfort, benefits to the subject, confidentiality of records, right to refuse participation and terminate participation in the study at any time. The informed verbal consent was obtained from the respondents after explaining the purpose of study. Participants were assured of confidentiality with regard to all information acquired.

## 4. Results

### 4.1. Socio-demographic characteristics of study participants

From 422 HCWs selected four hundred thirteen 413 (98%) healthcare professionals were participated in this study. According to this study, the most frequent respondents 197(47.7%) were aged between 25–29 years old with mean value of 27.4 and SD of 3.75 ranging from 20 to 48 years. From overall respondents 233 (56.4%) were male. In this study majority of respondents were single 221(53.5%). From HCWs the largest numbers of respondents were nurses 171 (41.4%) and the smallest numbers of respondents from health professionals were Anaesthesia 8 (1.9%). From the description, majority of HCWs had work experience 6–9 years 130 (31.5%) with mean value of 5.38 and SD of 4.5 ranging from 1 to 21 years.

### 4.2. Level of compliance with standard safety precaution

#### 4.2.1. Hand hygiene

According to this study, from the HCWs 78(18.9%) always wash hands before touching a patient and 109 (26.4%) of HCWs always wash hands before clean/aseptic procedures. Among the respondents of HCWs 320(77.5%) always wash hand after touching body fluid exposures and 281(68%) of HCWs always wash hand after touching a patient. From HCWs participated in this study, 134(32.4%) always wash hands immediately after removal of gloves, 112(27.1%) always wash hands between patient contact and 110(26.6%) always wash hands touching patient surroundings. The findings are presented in Table 1 below.

**Table 1:** Level of compliance with hand hygiene among healthcare workers of Hawassa comprehensive specialized hospital, 2019.

Compliance variable			Response (%)		
	Never (%)		Sometimes (% )	Always (%)	
I wash hands before touching a patient	253 (61.3)		82 (19.9)	78 (18.9)	
I wash hands before clean/aseptic procedures	232 (56.2)		72 (17.4)	109 (26.4)	
I wash hand after touching body fluid exposures	26 (6.3)		67 (16.2)	320 (77.5)	
I wash hands after touching a patient	46 (11.1)		86 (20.8)	281 (68)	
I wash hands immediately after removal of gloves	194 (47)		85 (20.6)	134 (32.4)	
I wash hands between patient contact	231 (55.9)		70 (16.9)	112 (27.1)	
I wash hands touching patient surroundings	213 (51.6)		90 (21.6)	110 (26.6)	

Compliance with personal protective SPs among HCWs of Hawassa comprehensive specialized Hospital is presented in the Table 2 blow.

**Table 2:** Compliance with personal protective safety precautions among healthcare workers of Hawassa comprehensive specialized hospital, 2019.

Compliance variable	Never (%)	Sometime (% )	Always (%)
I protect myself against body fluids of all patients regardless of their diagnosis	35 (8.5)	85 (20.6)	293 (70.9)
I provide care considering all patients as potential infectious	228 (55.2)	98 (23.7)	87 (21.1)
I wear clean gloves whenever there is possibility of any body fluids	115 (27.8)	60(14.5)	238 (57.6)
I avoid wearing my gown out of work place	79 (19.1)	55 (13.3)	279 (67.6)
I wear eye goggles when indicated	249 (60.3)	70 (16.9)	94 (22.8)
I wear mask when indicated	220 (53.3)	87 (21.1)	106 (25.7)
I wear boots when indicated	229 (55.4)	73 (17.7)	111 (26.9)

**Fig 1:**
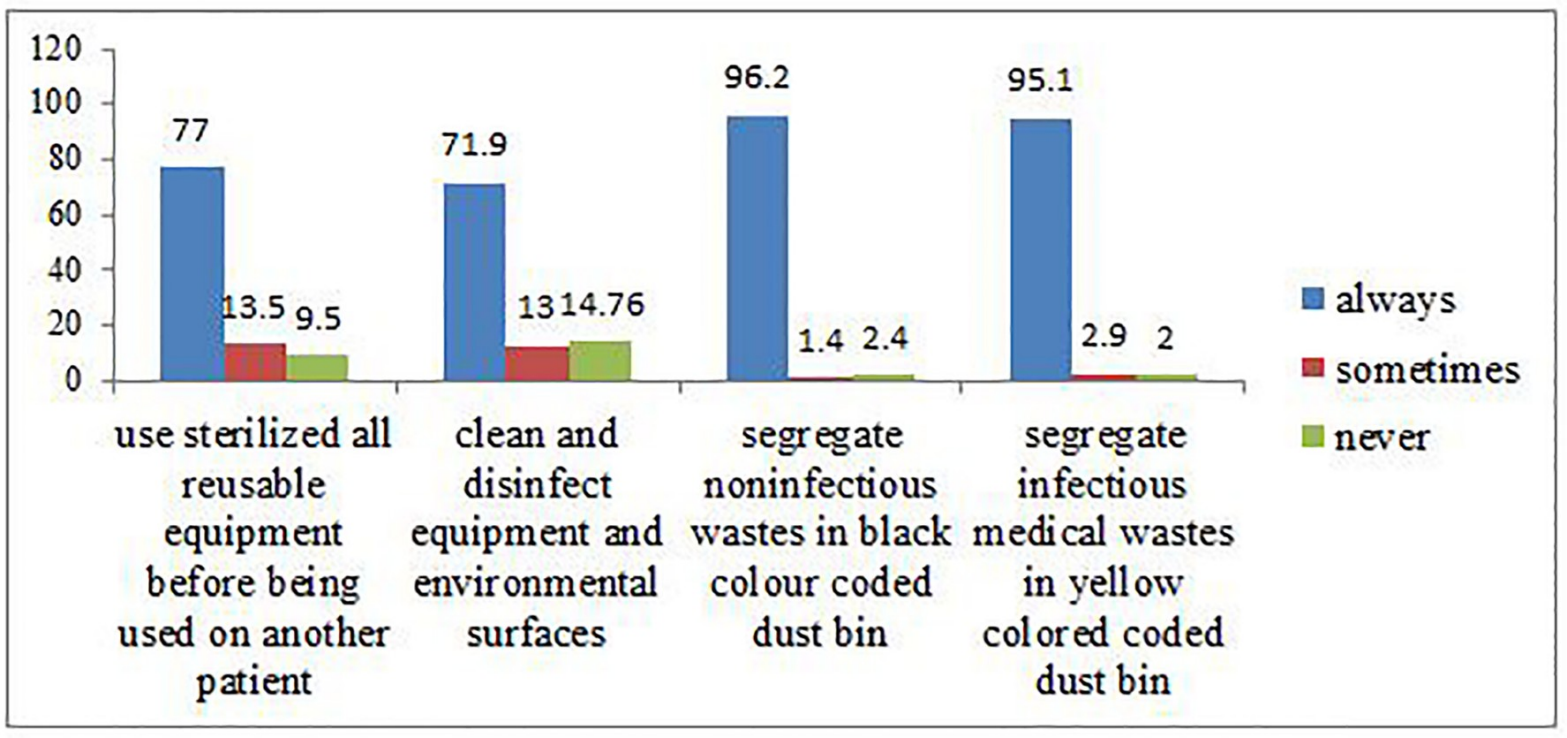
Frequency of healthcare waste management within HCWs in Hawassa comprehensive specialized hospital.

Frequency of healthcare waste management within healthcare workers (HCWs) in Hawassa comprehensive specialized Hospital is presented in Fig 1.

Level of compliance with healthcare waste management and sharp safety precautions among HCWs of Hawassa comprehensive specialized Hospital is presented in the Table 3 below those variables significantly associated were analyzed together in the multi-variable analysis by using backward logistic regression only sex, marital status, accessibility of sharp waste collection container, availability of running tape water in workroom, training and conducted internal infection prevention and control supportive supervision in the facility were statistically significant factors of compliance with standard safety precaution as illustrated in Table 4 below. In this study, female HCWs were 2.76 times more likely to be compliant with standard SP when compared with male HCWs. HCWs who had married were 4.2 times more likely compliant with standard SP when compared with single HCWs. HCWs who had accessibility of safety box 3.4 times more likely always compliant with standard SP. HCWs whose work room had available running tape water were 2.68 times more likely compliant when compared with work room had not available running tape water HCWs. HCWs who had perceived training on standard SP were 3.99 times more likely comply with standard SP when compared with not perceived training on standard SP HCWs. HCWs who had internal IPC supportive supervision were 5.8 times more likely to compliant with standard SP when compared with not had internal IPC supportive supervision HCWs.

**Table 3:** Level of compliance with healthcare waste management and sharp standard safety precautions among HCWs of Hawassa comprehensive specialized hospital, 2019.

Compliance variables	Never (%)	Sometime (% )	Always (%)
I use sterilized all reusable equipment before being used on another patient	40(9.7)	56(13.6)	31(76.8)
I segregate noninfectious wastes in black colour coded dust bin	10(2.4)	6(1.5)	397(96.1)
I clean and disinfect equipment and environmental surfaces	61(14.8)	55(13.3)	297(71.9)
I segregate infectious medical wastes in yellow colored coded dust bin	8(1.9)	12(2.9)	393(95.2)
I dispose immediately all used needles and syringes in to safety box	0	12(2.9)	401(97.1)
I place used sharps in puncture-resistant container at point of use	6(1.5)	0	407(98.5)
I recap needles	303(73.4)	65(15.7)	45(10.9)
I bend needles	370(89.6)	33(8)	10(2.4)

**Table 4:** Multivariable analysis result for factors affecting compliance with standard safety precautions in Hawassa comprehensive specialized hospital, 2019.

Variables		Noncompliant	compliant	AOR (CI 95%)	p-value
Sex	Female	112	68	2.76(1.34–5.54)	**0.004**
	Male	207	26	1	
Marital status	Married	113	79	4.2(2, 9.03)	**0.001**
	Single	206	15	1	
Safety box placed at hand reached areas in workroom	Yes	103	72	3.4(1.6, 7.17)	**0.001**
	No	216	22	1	
Have you ever taken IP training	Yes	155	81	3.99(1.46, 10.9)	**0.007**
	No	164	13	1	
Availability of running tape water in the room of you assigned	Yes	144	81	2.68((1.15, 6.2)	**0.02**
	No	175	13	1	
Think there is lack of commitment on the part of HF to invest IP	Yes	188	69	1.87(0.9, -3.85)	0.87
	No	131	25	1	
Have you ever participated in any training program about standard SP	Yes	210	86	3.7(1.29,10.9)	**0.015**
	No	109	8	1	
Does hospital provide training on IP upon hire	Yes	159	69	3.2(1.59, 6.5)	**0.001**
	No	160	25	1	
Have ever conducted internal IPC supportive supervision in this facility	Yes	116	84	5.8(2.54, 13.48)	**0.001**
	No	203	10	1	

Hosmer and Lemeshow Test = 0.948 therefore the model adequately fits the data.

## 5. Discussion

The study finding revealed that over all compliance with standard SP among HCWs was only 56.5%. It was very similar when compared with study conducted in two teaching hospitals of Gondar and Felege hiwot in Amhara region were 55% [14] and low when compared with study conducted in Addis Ababa were 66.1% [12]. Although it was high when compared with Wolayta zone were 42.4% and Mizan Tape general hospital 46.8% [15, 16]. Difference in compliance with standard SPs could be difference in type of healthcare facilities from which HCWs were selected to participate in the study, sampling technique, study setting and HCWs experiences.

HCWs always washing hand before touching a patient were only 18.9%. HCWs always washing hands before clean/aseptic procedures were 26.4%. HCWs always washing hands immediately after removal of gloves were 32.4% and always washing hands between patients contacts were only 27.1%. This finding was similar with the study in Kenya in which hand washing between tasks and between patients were only 15.5% and 20% respectively.

HCWs always washing hand before touching a patient were only 18.9%. HCWs always washing hands before clean/aseptic procedures were 26.4%. HCWs always washing hands immediately after removal of gloves were 32.4% and always washing hands between patients contacts were only 27.1%. This finding was similar with the study in Kenya in which hand washing between tasks and between patients were only 15.5% and 20% respectively. And also similar study in Gondar comprehensive specialized hospital showed in HCWs always washing hand before touching a patient was 18.2%, HCWs always washing hands between patient contact was 19.4% and always washing hands after touching patient surroundings was 22.4% [6, 9]. This might be due to workload, unavailability and accessibility of hand washing facility and continuous running tape water in the health facility.

Regarding PPEs, this study declared that HCWs always protect themselves against body fluids of all patients regardless of patients’ diagnosis were 70.9%. The findings on the number of HCWs expressed low compliance with always wear a waterproof apron whenever there is a possibility of body fluid splashing in their body was only 30.5%, always wear boots whenever there is the potential of the legs coming in to contact with blood body fluids and other contaminated materials was only 26.9%, wear mask whenever there is possibility of transmission of pathogens through close respiratory or mucous contact with respiratory secretions was only 25.7% and wear eye goggles whenever there is a possibility of body fluid splashing in their face was only 22.8%. These finding were consistent with study conducted in tertiary care hospital in South India in which HCWs always wear eye goggles when indicated were 22% and also Sao Paulo in psychiatry hospitals in which HCWs expressed low compliance with aprons, goggles and protective masks [17, 18]. These similarities might be due to shortage of PPE, work load and discomfort or unfitness of Personal protective equipment. Sharp safety precautions had showed highest compliance among HCWs in this study. As the study reported, HCWs always dispose immediately all used needles and syringes in to safety box and place used sharps in puncture-resistant container at point of use were 97.1% and 98.5% respectively in this study. This finding is similar with study conducted in Addis Ababa in which HCWs performed SPs more than 90% [19]. This similarity might be as result of availability of SPs materials and increased perception of risk within HCWs.

The present study found out significant association in the compliance of among HCWs who had perceived training on standard safety precautions 3.99 times more likely compliance with standard SPs when compared with HCWs that did perceived training on standard SP. It is consistent with study in West Arsi district and Wolayta zone in which HCWs that had infection prevention training were five times more likely practice safely and two times more likely exposed to blood, body and fluids when compared with their counterparts respectively [15, 16]. Training has the effective influence on the behaviour and readiness of HCWs for performing activities based on the IPC guidelines and it increases the awareness and knowledge of HCWs towards complying standard SPs as different literatures indicated [18, 19].

It is the responsibility of health facility management to supervise and monitor regularly staff members their duties and compliance of standard SPs all the times at work place. This study revealed that HCWs whose assigned place of work supportive supervision performed on IPPC were 5.8 times more likely compliance with standard SPs when compared with IPPC supportive supervision not performed HCWs.

## 6. Conclusion and recommendations

Compliance with standard safety precautions requires special attention as result of the risk posed by the presence of infectious pathogens that cause serious negative impact on health of patients, visitors, attendants and healthcare workers. From our study, the overall level of compliance with standard safety precautions among healthcare workers considered to be very low. Factors such as healthcare workers being female, married, accessibility of safety box, availability of running tape water, participations in training program and health facility providing training upon hire and internal infection prevention and control supportive supervision were independent predictors of compliance with standard safety precautions.

Preparing and providing programmed continuous on job and up on hire training on compliance with standard safety precautions should be very crucial. The healthcare facility should fulfill personal protective equipment like mask, boots, aprons and eye goggles. Safety materials such as availing and accessing safety box and running tape water in work department should be valuable and necessary interventions for improvement of HCWs towards compliance with standard safety precautions. Healthcare workers should give attention for their hand hygiene compliance before and between any practices in healthcare facility. In addition to this, healthcare workers should provide care considering every patient as potential for transmission and acquiring of infectious agent. There should be strong consistent internal infection prevention and control supervision recommended for healthcare workers.

## Abbreviations

CASH: Clean and safe health
CDC: Centers for disease prevention and control
EHAQ: Ethiopia hospitals alliance for quality
EHRIG: Ethiopia hospitals reform implementation guidelines
FMOH: Federal Ministry of Health
HCAIs: Health Care Associated Infections
HCWs: Health care workers
HF: Health facility
HIV: Human immunodeficiency virus
IPPC: Infection prevention practices and control
PPE: Personal protective equipment
SPs: Standard precautions
SPSS: Statistical Package for Social Science
WHO: World health organization
